# Percutaneous retroperitoneal splenorenal shunt creation after failed PVR-TIPS attempt

**DOI:** 10.1186/s42155-026-00663-1

**Published:** 2026-02-28

**Authors:** Omari Christie, Bryan Nicholas Swilley

**Affiliations:** Department of Radiology, Division of Interventional Radiology, Emory University, 1364 Clifton Road Suite AG-05, Atlanta, GA 30322 USA

**Keywords:** TIPS, PVR-TIPS, PRESS, Variceal hemorrhage, Portal hypertension

## Abstract

Gastric and esophageal variceal bleeding causes significant morbidity and mortality. TIPS creation is one of many methods to treat and reduce the risk of variceal hemorrhage. In such cases where TIPS cannot be created, percutaneous retroperitoneal splenorenal shunt (PRESS) creation serves as one alternative to decompress the portal venous system. This case highlights a case of PRESS creation from splenic vein to left adrenal vein as a method to decompress the splanchnic venous system in cases of chronic portal vein thrombosis with cavernous transformation when PVR-TIPS fails, with subsequent 6 month follow-up.

## Background

Upper gastrointestinal bleeding remains a significant cause of morbidity and mortality in patients with portal hypertension. Approximately 50% of patients with cirrhosis develop gastroesophageal varices [1]. Gastric varices cause approximately 10–30% of variceal hemorrhage. Thirty-five to 90% of gastric varices rebleed after initial hemostasis [2]. TIPS creation provides initial hemostasis in up to 90% of patients with bleeding gastric varices, and pre-emptive TIPS after initial endoscopic control of variceal hemorrhage has shown a 25% absolute risk reduction in mortality [3, 4]. TIPS and BRTO performed together can further reduce the risk of rebleeding [5]. Chronic portal vein occlusion can add technical complexity to TIPS creation, but PVR-TIPS can be performed successfully in up to 98% with advanced techniques [6]. For cases of bleeding gastroesophageal varices that are not candidates for PVR-TIPS, PRESS has been described as a new technique to create an extrahepatic portosystemic shunt [7–9].

## Case presentation

A 62 year old male with history of Child Pugh A cirrhosis complicated by portal vein thrombosis with cavernous transformation on Eliquis for 7 years initially presented to an outside hospital with bleeding gastric and esophageal varices. Endoscopy revealed grade 3 esophageal varices, large gastric varices and portal hypertension gastrostomy. The outside facility attempted TIPS creation, unsuccessfully. The patient was felt to have no reasonable surgical or interventional radiology options at the outside facility, so he was then referred to our facility for TIPS creation. At the time of presentation to our facility, he was Child Pugh A. MELD 8. BMI 27.4 kg/m2.

After PVR-TIPS was discussed at a clinic visit, attempted PVR-TIPS was unsuccessful. Options of partial splenic embolization and/or percutaneous retroperitoneal splenorenal shunt (PRESS) creation were discussed. He elected to try PRESS with partial splenic embolization as a last resort.

Careful pre-procedure review of cross-sectional imaging revealed proximity of the main splenic vein to a spontaneous gastro-adrenal shunt. The spontaneous gastro-renal shunt required blood to pass through and pressurize gastric varices. This was chosen as the point of target from the splenic vein to the renal vein. Written informed consent was obtained before the procedure.

Trans-splenic access was obtained using a Greb access set, with subsequent exchange of the Greb catheter for a 6 French vascular sheath. Left common femoral arterial and venous access were also obtained, with 6 French vascular sheath placements into each. A SOS catheter was used to access the celiac access and advanced into the splenic artery for visualization of the splenic artery during splenorenal shunt creation. A Simmons 2 catheter was used in the right common femoral vein sheath to select the left renal vein and then the left adrenal vein spontaneous gastro-renal shunt outflow. Over a Rosen wire, the Simmons 2 catheter and vascular sheath were exchanged for an 8.5 French × 55 cm Bayliss Passport steerable sheath was advanced into the left renal vein for increased stability. A 20 mm Gooseneck snare was then positioned within the gastro-renal shunt outflow through the Passport sheath.

Next, Cobra 2 catheter was used through the trans-splenic access to target the snare with a Bayliss radiofrequency guidewire under fluoroscopic guidance. After securement of the radiofrequency wire with the snare, it was pulled through the common femoral vein sheath for body floss. A 4 French Terumo Navicross catheter was then advanced across the radiofrequency wire, and the radiofrequency wire was exchanged for the exchange length Rosen wire. Via the common femoral vein access sheath, a 9 mm × 10 cm Viabahn stent graft was positioned and deployed across the splenorenal shunt tract. The splenorenal shunt was then post-dilated with an angioplasty balloon. Venogram through the sheath and delayed splenic arteriogram demonstrated patent flow across the new splenorenal shunt. Pressure measurements were not performed in this case due to technical difficulties with the transducer equipment.

The splenic access tract was closed with a 5–7 mm MVP vascular plug and 1:1 lipiodol:glue mixture. The common femoral arterial access was closed with Angioseal, and hemostasis of the left common femoral vein access was achieved with manual compression.

**Fig. 1:**
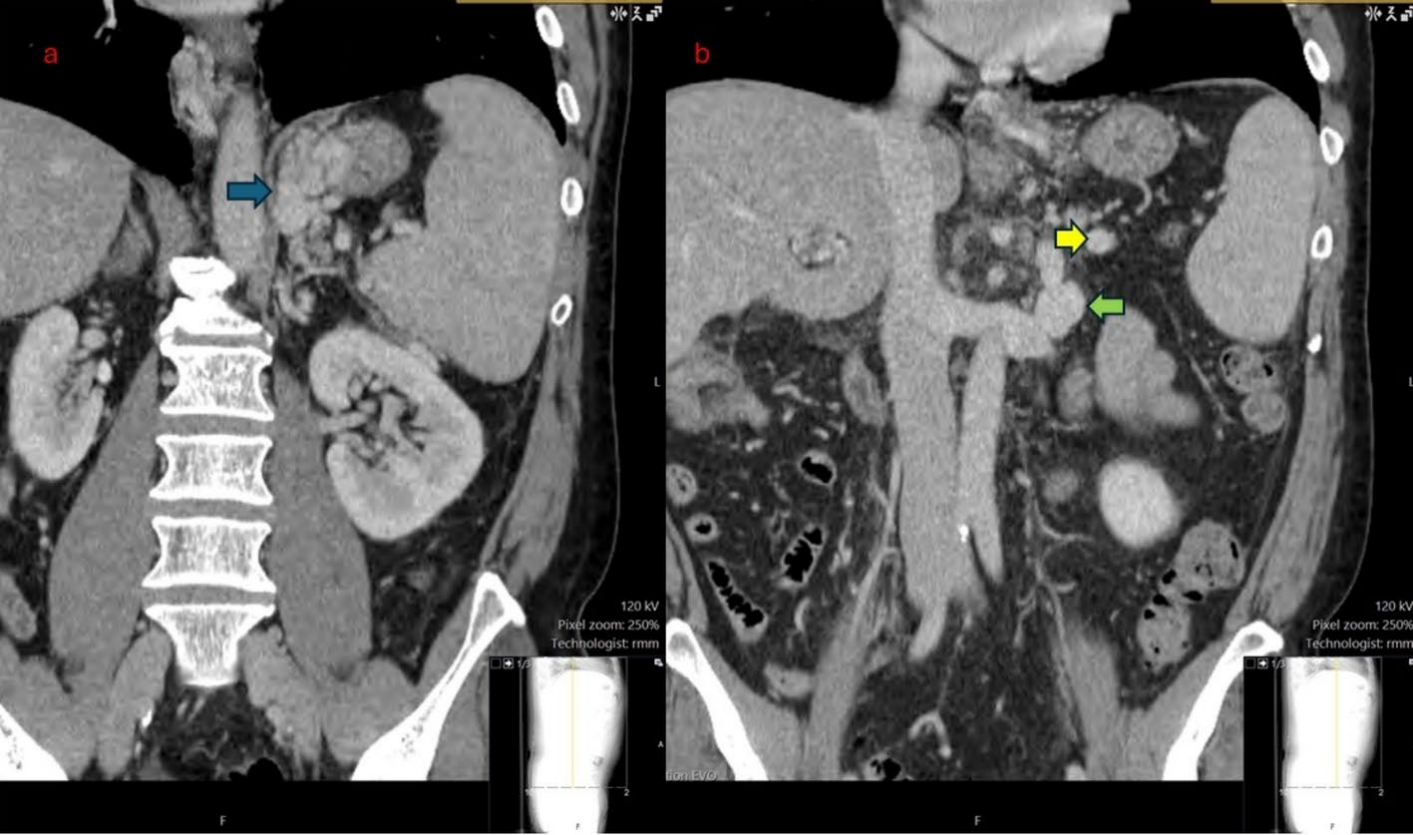
**a** demonstrates large gastric varices (blue arrow). **b** shows proximity of splenic vein (yellow arrow) to outflow of spontaneous gastro-adrenal shunt (green arrow)

**Fig. 2:**
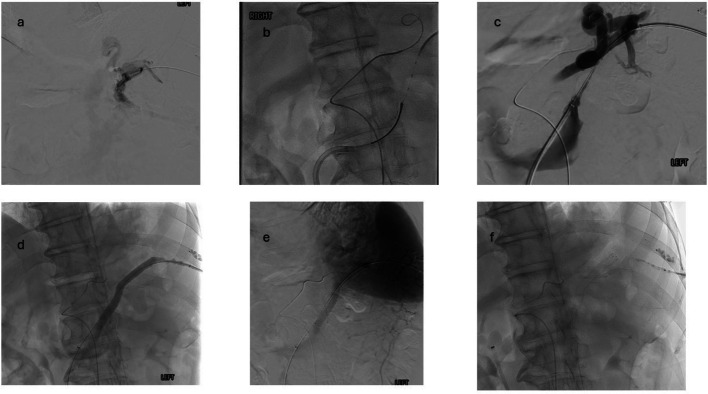
**a** splenic venogram after initial trans-splenic access. **b** The power wire passing from the Cobra 2 catheter within the splenic vein to the snare within the gastro-adrenal shunt outflow. **c** demonstrates simultaneous venogram through trans-splenic sheath and left common femoral vein sheath to show the shunt length. **d** and **e** Patency of the stent after deployment of the Viabahn stent within the shunt tract. **f **The MVP plug and glue tract embolization from this procedure and the prior PVR-TIPS attempt

**Fig. 3:**
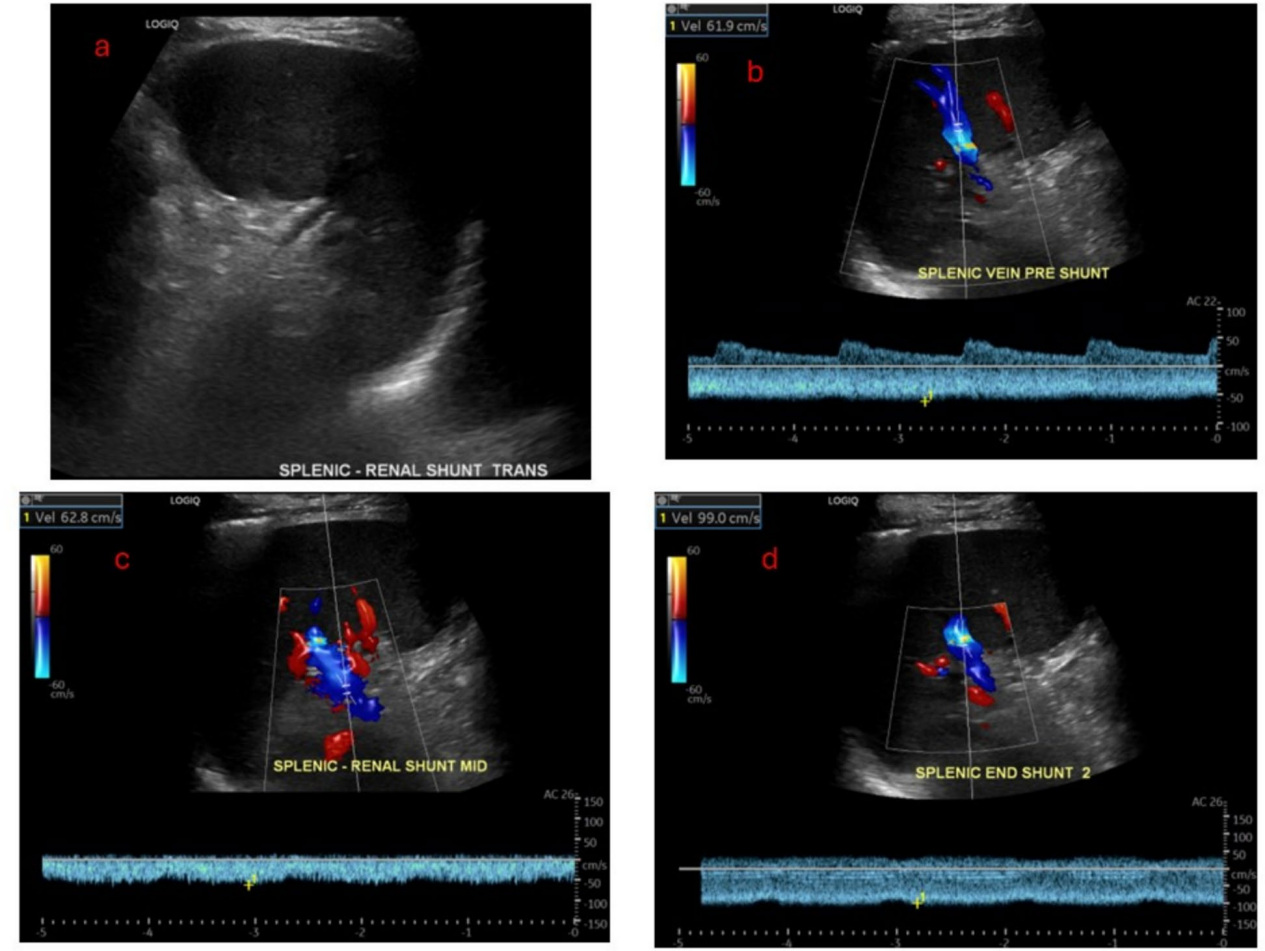
The figure demonstrates doppler ultrasound at 2 months post-PRESS creation. **a** Grayscale image of the spleen and splenorenal shunt. **b**-**d** Patent color and spectral doppler flow within the splenic vein and splenorenal shunt

**Fig. 4:**
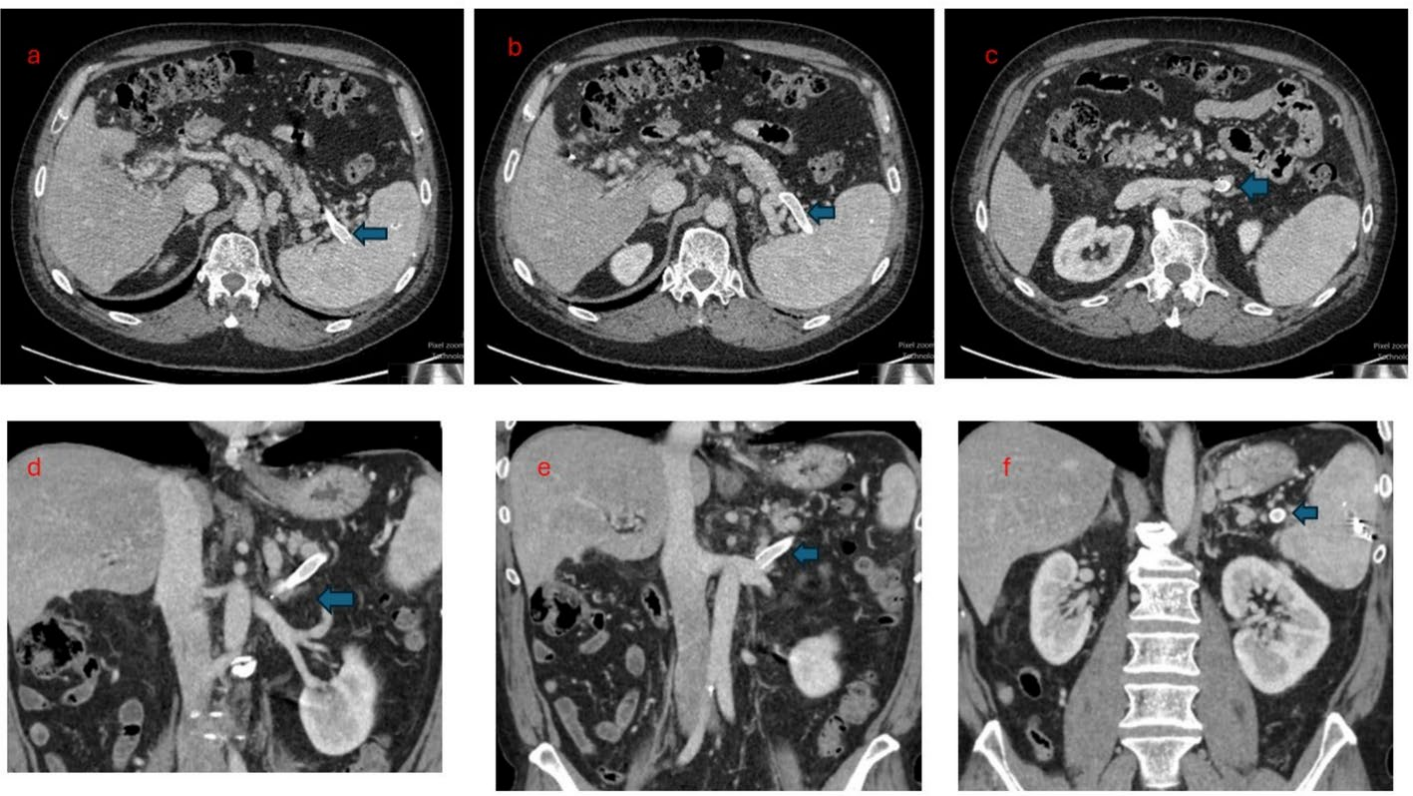
The figure demonstrates 6 months patency of the splenorenal shunt (blue arrow)

The patient was then transported to the intensive care unit for post-procedure observation. That evening, he reported feeling great and was able to get out of bed. He was discharged three days later. He reported doing well at his two month follow up visit, and doppler ultrasound at that time demonstrated patency of the splenorenal shunt. At 6 months, he still felt well, and CT with IV contrast demonstrated continued patency of the splenorenal shunt. At the 6-month clinic visit, he still had not experienced recurrent gastrointestinal bleeding (Figs. 1, 2, 3, and 4).

## Conclusions

Although surgical splenorenal shunt creation has existed since 1947 and has been studied extensively, percutaneous retroperitoneal splenorenal shunt creation provides a novel approach for decompression of portal hypertension in patients who are not candidates for TIPS or PVR-TIPS [10]. Much of the existing literature about PRESS lacks long-term follow up. To our knowledge, this is only the third report of PRESS to contain long-term follow up, with the other cases having splenic vein to left renal vein shunts. Frenk et al. demonstrated that the shunt can be successfully created from splenic vein to left adrenal vein, however their patient failed to recover from initial bleeding episode and died nine days after the procedure [8]. This report demonstrates a case in which radiofrequency wire-assisted PRESS has been well tolerated and remained patent for 6 months in a shunt created from splenic vein to left adrenal vein. PRESS remains an interesting type of extrahepatic porto-systemic shunt creation that merits additional research as a possible alternative in cases when TIPS cannot be created.

## Data Availability

Not applicable.

## Abbreviations

TIPS: Transjugular intrahepatic portosystemic shunt
PVR-TIPS: Portal vein recanalization with transjugular intrahepatic portosystemic shunt
PRESS: Percutaneous retroperitoneal splenorenal shunt
